# Differential circadian time modulates of dentate gyrus and CA1 excitation-inhibition balance and seizure threshold

**DOI:** 10.1186/s13041-026-01346-7

**Published:** 2026-08-28

**Authors:** Rongjing Ge, Menghan Zhang, Daoming Cheng, Bingjing Sun, Hong Ni, Hui Zhang, Jianhong Liu, Hao Qian, Bei Liu, Jiandong Yu

**Affiliations:** 1 Department of Neurosurgery, The First Affiliated Hospital of Bengbu Medical University, Bengbu, Anhui China; 2 Laboratory of Brain and Psychiatric Disease, Bengbu Medical University, Bengbu, Anhui China; 3 Anhui Engineering Research Center for Neural Regeneration Technology and Medical New Materials, Bengbu Medical University, Bengbu, Anhui China; 4 Sichuan Provincial Key Laboratory for Human Disease Gene Study and the Center for Medical Genetics, Department of Laboratory Medicine, Sichuan Academy of Medical Sciences and Sichuan Provincial People’s Hospital, School of Medicine, University of Electronic Science and Technology of China, No. 4, Section 2, North Jianshe Road, Chengdu, Sichuan China; 5 School of Basic Medicine, Bengbu Medical University, No. 287, Changhuai Road, Bengbu, Anhui China

**Keywords:** Circadian rhythm, Optogenetic, Temporal lobe epilepsy, Intrinsic excitability, Synaptic transmission, EEG recording

## Abstract

Circadian rhythms regulate seizure threshold and neuronal excitability in epilepsy. Temporal lobe seizures tend to arise more frequently during the diurnal phase relative to the nocturnal phase, yet dentate gyrus (DG) granule cells display elevated intrinsic excitability and CA1 pyramidal neurons show enhanced synaptic excitability at night. Here, we used male adult ICR mice (8–10 weeks old) as experimental subjects. Systemic pilocarpine injection was applied to pharmacologically trigger acute seizures, while AAV (pAAV2/9-CaMKIIα-hChR2 (H134R) - EYFP) delivery followed by optogenetic stimulation (10 Hz, 20 ms pulse width, 5 mW 473 nm blue light) was implemented to evoke region-specific hippocampal seizures. Combining continuous video-electroencephalography (vEEG) recording, optogenetically induced seizure assays, mRNA sequencing, and whole-cell patch-clamp electrophysiology, we investigated circadian modulation of neuronal excitability and seizure threshold within the DG and CA1 subfields of the hippocampus. Our results revealed divergent circadian regulation of seizure thresholds between the DG and CA1 regions. Notably, the diurnal-nocturnal seizure threshold pattern in CA1 recapitulated that seen in systemic pilocarpien-provoked seizure models. We further confirmed that circadian cycles exert distinct regulatory effects on excitatory/inhibitory synaptic transmission and the intrinsic excitability of hippocampal principal neurons in the DG and CA1. Collectively, this work highlights region-specific circadian control over seizure susceptibility and the brain excitation-inhibition balance. Elucidating circadian influences on hippocampal physiology and seizure threshold will improve the translational value of anti-epileptic therapies and guide time-stratified seizure intervention strategies.

**Supplementary Information:**

The online version contains supplementary material available at https://doi.org/10.1186/s13041-026-01346-7.

## Introduction

Epilepsy is a prevalent neurological disorder characterized by an imbalance between neuronal excitation and inhibition [1]. Despite the development of numerous anticonvulsant medications aimed at modulating synaptic transmission and neuronal excitability, a subset of patients with epilepsy exhibits resistance to these commonly prescribed drugs [2, 3]. Temporal lobe epilepsy, in particular, is recognized as a form of drug-resistant epilepsy. Consequently, it is imperative to conduct further investigations into the mechanisms underlying drug-resistant epileptic seizures and to identify potential therapeutic targets for the treatment of epilepsy.

Both human and rodent seizures exhibit a distinct circadian rhythm. Frontal lobe epilepsy predominantly manifests during sleep, whereas generalized seizures are more frequently observed during wakefulness and daylight hours [4]. Chronic seizures in rodent models are more likely to occur during the diurnal phase compared to nocturnal phase [5–7]. Furthermore, the induction of temporal lobe seizures in rodents through the administration of kainic acid (KA) and electrical stimulation has been shown to result in a higher seizure threshold during nocturnal hours [8, 9]. These findings suggest that circadian rhythm significantly influences the occurrence of seizures in temporal lobe epilepsy.

The onset of temporal lobe epilepsy primarily originates from the hippocampus, which encompasses the dentate gyrus (DG), CA1, CA2, CA3, and subiculum. Among these regions, the DG and CA1 have been the focus of extensive research regarding their roles in epileptic seizures [10, 11]. Despite the significant differences in function, neuronal types, and other characteristics between the DG and CA1, both regions are influenced by circadian rhythms. The intrinsic excitability of dentate gyrus granule cells (DGGC) is modulated by the expression of the gene *Bmal1*, which exhibits relatively high excitability during the nocturnal phase [12]. Additionally, the state of synaptic transmission and synaptic plasticity in CA1 pyramidal neurons is also regulated by circadian rhythms, demonstrating a higher excitation-inhibition ratio at nocturnal phase [13–15]. These findings indicate that the principal neurons within the hippocampus may exhibit elevated excitability during the nocturnal phase; however, temporal lobe epilepsy is characterized by a higher seizure threshold during this same period. These findings indicate that a detailed analysis of circadian rhythm modulation on neuronal synaptic transmission and excitability is needed to understand the initiation and progression of seizures.

Here, using male adult ICR mice aged 8–10 weeks, we employed optogenetics, brain slice patch-clamp recordings, and additional experimental approaches to investigate the relationship between circadian rhythms and the excitability of principal neurons across distinct hippocampal subregions, and to further explore correlations between temporal lobe epilepsy seizure threshold and the excitability of these key hippocampal principal neurons.

## Materials and methods

### Animals

In all experiments, ICR male mice aged 8 to 9 weeks were maintained under a 12-hour light/dark cycle, with illumination commencing at 7:00 a.m. and ceasing at 7:00 p.m. The animals had unrestricted access to food and water under standard environmental conditions, maintained at a temperature range of 21 to 26 °C.

All behavioral experiments in this study were conducted strictly in accordance with the guidelines and regulations of Laboratory Animal Ethics Committee of Bengbu Medical University and the ARRIVE guidelines. All experiments protocol using animals were approved by the Laboratory Animal Ethics Committee of Bengbu Medical University (Approval No. [2025] 590 and 597).

### Virus injection and optical fiber implantation

Mice were anesthetized using pentobarbital administered intraperitoneally at a dosage of 75 mg/kg [16] and their heads were secured in a stereotaxic apparatus (Shenzhen RWD Life Technology Co., Ltd.). Throughout the surgical procedure, the mice were maintained at an appropriate temperature on a heating pad while under anesthesia. A viral vector containing ChR2 EYFP under the CaMKII α promoter (200 nL, pAAV2/9-CaMKIIα-hChR2 (H134R) - EYFP, titer 2.2 × 10^13 viral genomes/mL) was injected at the following coordinates: dentate gyrus (DG) (anterior-posterior (AP) -1.82 mm; medial-lateral (ML) ± 1.17 mm; dorsal-ventral (DV) -2.16 mm) and CA1 (AP -2.00 mm; ML ± 1.50 mm; DV -1.50 mm), selectively targeting putative glutamatergic neurons. Following the viral injection, an optical fiber was implanted 300 μm above the injection site [17] and secured to the skull using dental resin. The mice were then housed separately for duration of three weeks to allow for complete viral expression, after which histological verification of the fiber placement and viral expression was conducted following behavioral experiments.

### EEG recording

Electroencephalogram (EEG) signals were recorded from the surface of the mouse skull utilizing EEG acquisition devices. Following the administration of anesthesia, three micro-screws were implanted into the skull’s surface, designated as the positive electrode, negative electrode, and reference electrode, respectively. These electrodes were secured to the skull using dental resin. The coordinates for the insertion positions of the positive, negative, and reference electrodes are as follows: the left frontal lobe area (anterior-posterior (AP) = + 2.5 mm; medial-lateral (ML) = + 1.5 mm), the right parietal lobe area (AP = -2.5 mm; ML = -2.4 mm), and the right cerebellar area (AP = -5.5 mm; ML = -1.5 mm). All EEG recordings were performed utilizing a model 1700 4-channel amplifier (A-M systems, USA). The data were low-pass filtered at 500 Hz and high-pass filtered at 0.5 Hz, digitized using a DigiData 1440, and acquired with pCLAMP 10 (Molecular Devices, USA) at a sampling frequency of 1 kHz. Data analysis was conducted using Clampfit (Molecular Devices). A digital video camera was used to simultaneously monitor behavior during the EEG recordings.

### Pilocarpine induced acute seizure

During the diurnal phase (13:30, ZT6) and the nocturnal phase (21:30, ZT14), separate cohorts of mice were administered scopolamine (0.6 mg/kg) via intraperitoneal injection. Thirty minutes later, pilocarpine (285 mg/kg) was also administered intraperitoneally to induce acute seizures. Electroencephalogram (EEG) signals and video recordings were continuously captured for duration of 120 min to assess the severity and alterations in EEG signals during the seizure episodes.

### Light stimulation induced seizure

Two distinct time points were selected in accordance with our prior published work [9] for the experiment: the diurnal phase at 13:30 (ZT6) and the nocturnal phase at 21:30 (ZT14). A blue laser with a wavelength of 473 nm was employed, transmitted through a fiber optic with a diameter of 200 μm (Shenzhen RWD Life Technology Co., Ltd.). The laser power was calibrated to approximately 5 mW, as measured by a power meter. Subsequently, blue light (473 nm, 10 Hz, 20 ms pulse width, 5 mW) was utilized to induce seizures.

### Seizure behavior

Both pilocarpine administration and optogenetic activation have been shown to induce acute seizures in murine models, primarily presenting with symptoms such as salivation and limb twitching. All the mice were video-EEG recording for the seizure severity. The specific grading criteria for epileptic seizures are based on the modified Racine scale as proposed by Heather Shibley [18]. According to this scale, stage 1 is defined by tail stiffness; stage 2 is characterized by shaking reminiscent of a wet dog; stage 3 involves low-intensity twitching and spasms of the unilateral forelimb; stage 4 is marked by bilateral forelimb muscle spasms accompanied by standing; stage 5 entails a temporary loss of control over bilateral forelimbs and hind limb spasms, as well as compromised postural control; and stage 6 indicates death. Generalized convulsive status epilepticus (GS) was defined as stage 4 and stage 5 convulsive seizure episodes.

### Quantitative real-time PCR (qRT-PCR)

Hippocampal (DG and CA1) tissues were collected from control mice (10 mg tissue per animal). Total RNA extraction was performed using the FastPure Cell/Tissue Total RNA Isolation Kit V2 according to the manufacturer’s protocol. RNA concentration and purity were quantified spectrophotometrically (A260/A280 ratio).

Reverse transcription was conducted with the HiScript III All-in-One RT SuperMix Perfect for qPCR under the following thermal conditions: 50 °C for 15 min (cDNA synthesis) followed by 85 °C for 5 s (enzyme inactivation). Subsequent qPCR amplification utilized the ChamQ Universal SYBR qPCR Master Mix with the following cycling parameters: initial denaturation at 95 °C for 30 s, followed by 40 cycles of 95 °C for 10 s (denaturation) and 60 °C for 30 s (annealing/extension).

Relative mRNA expression levels were calculated using the 2^−ΔΔCT^ method, normalized to housekeeping genes. All reactions were performed in triplicate with appropriate no-template controls.

### RNA extraction

Total RNA was extracted from samples using TRIzol™ Reagent (Thermo Fisher Scientific) according to the manufacturer’s instructions. A small metal screw was added to the sample tube containing tissue homogenization buffer or TRIzol™, and the mixture was vortexed for 30 s at high speed. The tube was then spun for 1 min at maximum speed in a tabletop cold centrifuge, and the supernatant was collected for RNA extraction. To enhance phase separation during RNA extraction with TRIzol™ Reagent, 5PRIME Phase Lock Gel Heavy tubes (Quantabio) were utilized. RNA quantification was performed using a Nanodrop 2000 (Thermo Scientific) or the Quant-iT™ RNA Assay Kit (Thermo Fisher Scientific), following the manufacturer’s instructions.

### Library preparation and sequencing

Libraries were prepared using the TruSeq Stranded mRNA Kit (Illumina) according to the manufacturer’s instructions. Briefly, 500 ng of total RNA was poly-A selected using beads, fragmented through metal-ion hydrolysis, and converted into ds cDNA. Horizontal hippocampal slices with a thickness of 300 μm were sectioned using a vibratome (Leica VT1000P). The dentate gyrus (DG) and CA1 subregions were manually dissected from intact slices with a surgical scalpel and fine forceps. 100 ng of total RNA was utilized for library preparation due to limited material availability. The ds cDNA was end-repaired, adenylated, ligated with TruSeq CD indexes (Illumina) or IDT for Illumina-TruSeq UD indexes (Illumina), and subsequently amplified through 15 cycles of PCR. The libraries were quantified using the Quant-iT™ dsDNA HS Assay Kit (Thermo Fisher Scientific), pooled, and sequenced at the NGS Core Facility of the Salk Institute or at Novogene Co. The full list of evaluated genes is provided in Supplementary Table 1.

### Differential gene expression and pathway annotation analysis

The raw sequencing data underwent quality control and trimming using Trim Galore (version 0.6.10). Reads with suboptimal quality or lengths shorter than 30 base pairs were excluded from further analysis. The remaining high-quality reads were aligned to the mouse reference genome (GRCm39) using the STAR aligner (version 2.7.10b), with the “quantMode TranscriptomeSAM GeneCounts” option enabled for quantification. Differentially expressed genes (DEGs) were identified using DESeq2 (version 1.40.1), applying a threshold of a fold change greater than 0.15, as previously documented [19, 20]. Functional enrichment analysis was conducted through Gene Ontology (GO) term analysis using WebGestaltR (version 0.4.6) [21].

### Acute slice preparation

To obtain acute hippocampal slices, mice were anesthetized with 4% isoflurane and subsequently decapitated [22–24]. The brains were rapidly excised and immerged in a cutting solution composed of NaCl (124 mM), KCl (2.5 mM), NaH_2_PO_4_ (1.2 mM), NaHCO_3_ (24 mM), HEPES (5 mM), MgSO_4_ (2 mM), CaCl_2_ (0.5 mM), and sucrose (12.5 mM) at 4 °C, while being saturated with a gas mixture of 95% O_2_ and 5% CO_2_. Horizontal slices of 300 μm were prepared using a vibratome (Leica VT1000P). The slices were then incubated for 6 min at a temperature of 33.3 °C in an oxygenated N-Methyl-D-glucamine (NMDG) solution, which contained NMDG (110 mM), KCl (2.5 mM), NaH_2_PO_4_ (1.2 mM), NaHCO_3_ (25 mM), glucose (25 mM), MgSO_4_ (10 mM), CaCl_2_ (0.5 mM), and HCl (110 mM). Following this incubation, the slices were transferred to an oxygenated artificial cerebrospinal fluid (ACSF) solution consisting of NaCl (124 mM), KCl (2.5 mM), NaH_2_PO_4_ (1.2 mM), NaHCO_3_ (24 mM), HEPES (5 mM), MgSO_4_ (0.5 mM), and CaCl_2_ (2 mM) at the same temperature for a recovery period of 30 min. After recovery, the slices were maintained at room temperature.

### Whole-cell recording

Whole-cell recordings were conducted using patch pipettes with resistances ranging from 4 to 8 MΩ, filled with various internal solutions. For the recording of spontaneous inhibitory postsynaptic currents (sIPSCs), the patch pipette solution comprised the following components: KCl (40 mM), KGlu (110 mM), EGTA (0.5 mM), HEPES (10 mM), NaCl (4 mM), Mg_2_ATP (4 mM), TrisGTP (1 mM), and phosphocreatine (5 mM). The artificial cerebrospinal fluid (ACSF) used for perfusion contained kynurenic acid at a concentration of 3 mM. In contrast, for the recording of action potentials and spontaneous excitatory postsynaptic currents (sEPSCs), the patch pipette solution included KGlu (150 mM), EGTA (0.5 mM), HEPES (10 mM), NaCl (4 mM), Mg ATP (4 mM), TrisGTP (1 mM), and phosphocreatine (5 mM). All patch-clamp recordings were performed utilizing a MultiClamp 700B amplifier (Molecular Devices, USA). The data were low-pass filtered at 3 kHz, digitized using a DigiData 1550, and acquired with pCLAMP 10 (Molecular Devices, USA) at a sampling frequency of 10 kHz. Data analysis was conducted using Clampfit (Molecular Devices).

### Quantification and statistical analysis

Sample sizes (n = number of animals per group), the statistical tests employed, and the levels of statistical significance are detailed in the Figures and their corresponding Legends. Unless otherwise specified, values are presented as mean ± standard error of the mean (SEM). Behavioral assessments and quantification experiments were conducted in a blinded manner to eliminate bias. One-way or two-way analysis of variance (ANOVA) was utilized, followed by post hoc comparisons using the Bonferroni correction. An unpaired Student’s t-test was employed to compare two independent groups, while a paired t-test was used for the comparison of correlated or dependent variables. Mice that exhibited no viral expression were excluded from the final analysis. In the figures, asterisks denote the levels of statistical significance: *p < 0.05, **p < 0.01, ***p < 0.001, ****P < 0.0001. All statistical analyses were conducted using GraphPad Prism 5.0 software (GraphPad).

## Result

### The circadian rhythm modulates seizures induced by pilocarpine in mice

**Fig. 1:**
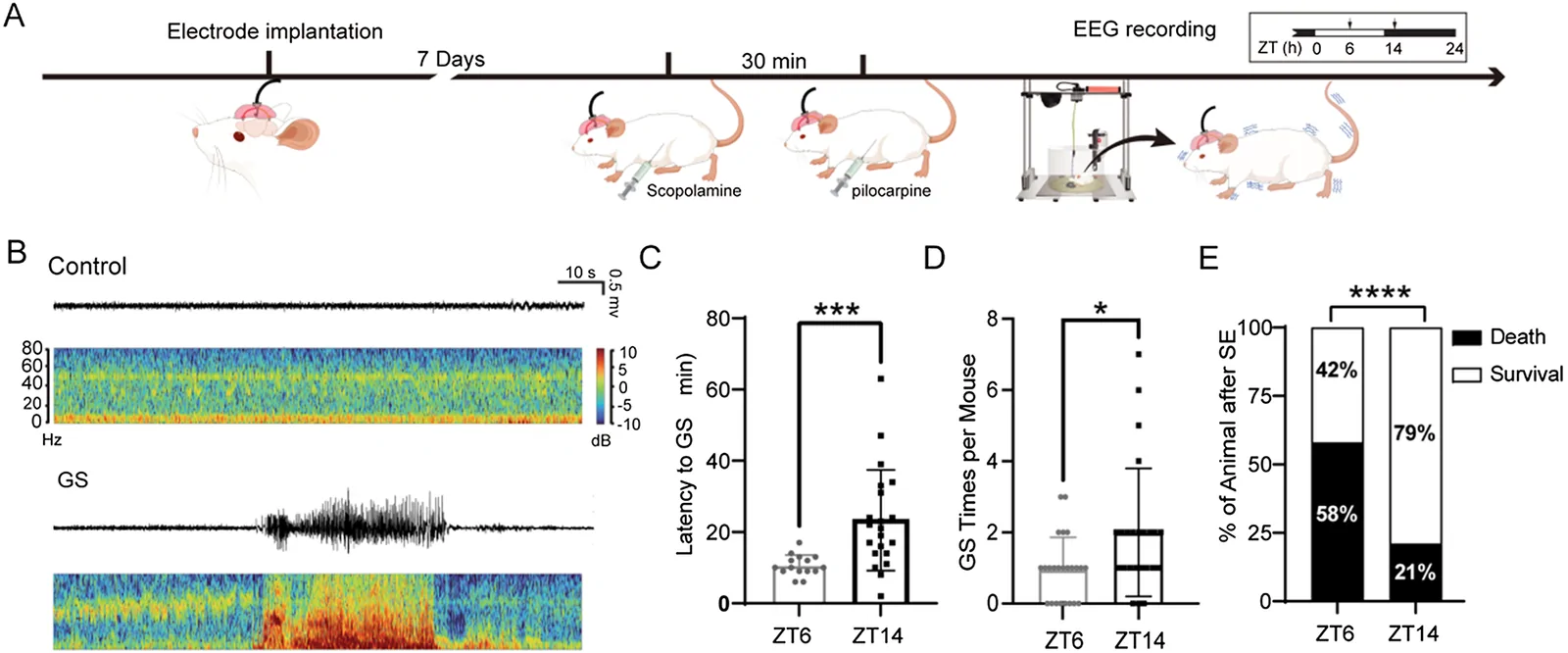
Circadian rhythms modulate pilocarpine-induced acute seizures. **A**: Experimental flowchart of the pilocarpine-induced acute epilepsy model. **B**: Typical EEG recordings and power spectrograms during behavioral seizures. Control EEG traces were acquired before mice administered pilocarpine injections. **C**-**D**: Latency to generalized seizures (**C**) and GS times (**D**) during diurnal and nocturnal phases, analyzed using unpaired t-tests (*P < 0.05, ***P < 0.001). **E**: the mortality rate of mice (chi-test, ****P < 0.0001). All data are presented as mean ± SEM. GS: generalized convulsive status epilepticus; ZT6: Zeitgeber Time 6; ZT14: Zeitgeber Time 14

To investigate the influence of circadian rhythm on seizure threshold, we administered pilocarpine to ICR mice to induce acute seizures (see Fig. 1A). Following the administration of pilocarpine, a pronounced epileptiform seizure was observed, characterized by a significant increase in voltage fluctuation amplitude—exceedingly twice the baseline—lasting approximately 30 s (see Fig. 1B). Pilocarpine was administered to induce seizures at two separate Zeitgeber time points representing the diurnal and nocturnal phases. The latency to generalized seizures (GS) was significantly prolonged in the nocturnal group relative to the diurnal group (Fig. 1C, ZT6: 14.4 ± 3.2 s; ZT14: 23.3 ± 2.9 s; 24 mice in each group. unpaired t-test, *P* = 0.014). In addition, mice exhibited substantially more GS episodes under nocturnal conditions (Fig. 1D, ZT6: 0.96 ± 0.19 seizures; ZT14: 2.00 ± 0.37 seizures; 24 mice in each group. unpaired t-test, *P* = 0.014). Mortality also differed markedly between timepoints: 14 out of 24 mice died at ZT6, whereas only 5 of 24 died at ZT14 (Fig. 1E, χ² test, *P* = 0.0002).

Collectively, these results demonstrate that pilocarpine requires a longer time to trigger generalized seizures at night, which is consistent with an elevated seizure threshold during the nocturnal phase. The higher GS count observed at ZT14 can be largely attributed to the drastically reduced mortality in nocturnal animals; most mice at ZT6 died after only one or two GS episodes and could not be tracked for additional seizure events.

### The circadian rhythms differentially modulate seizure thresholds in the dentate gyrus (DG) and CA1

**Fig. 2:**
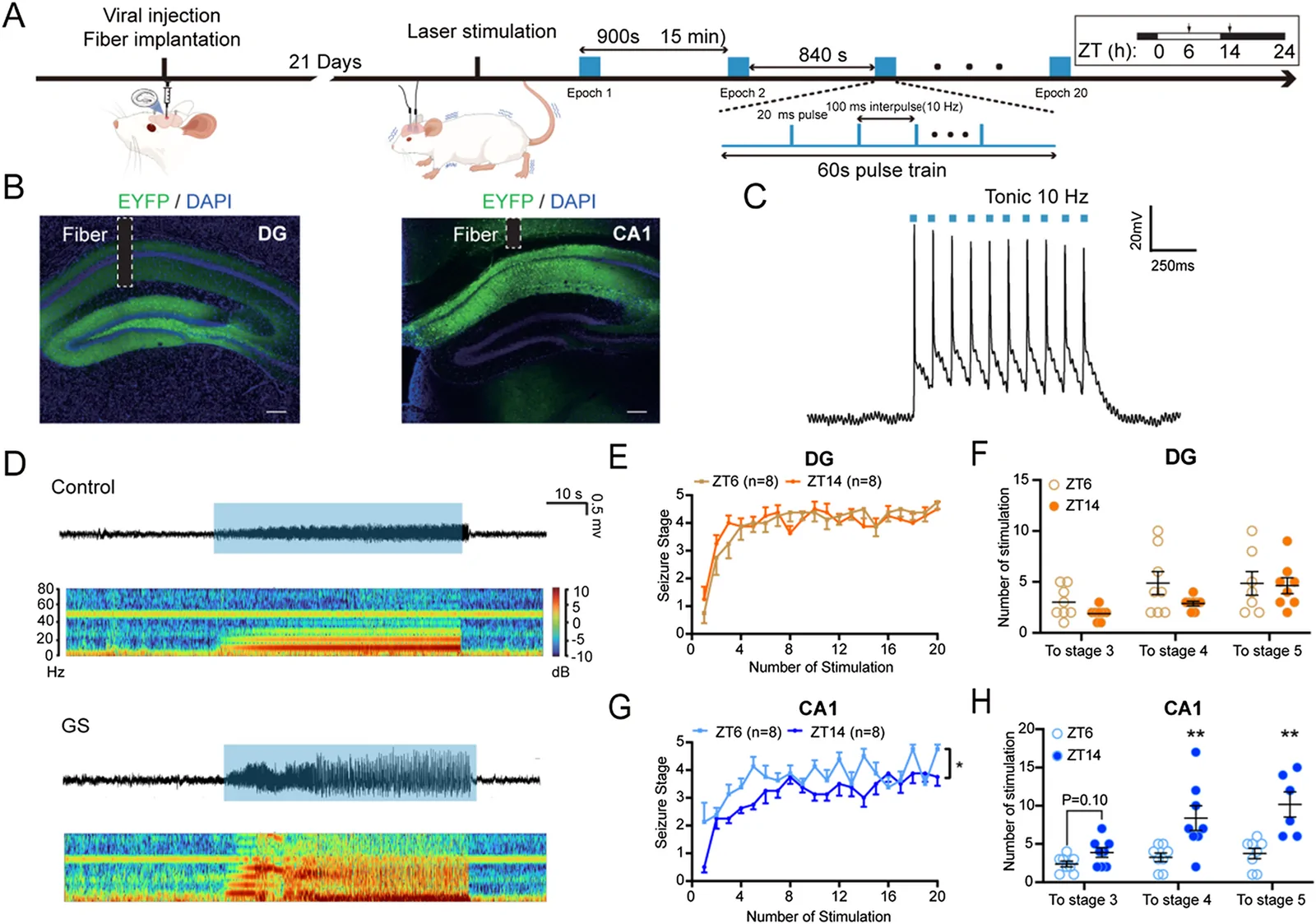
The circadian rhythm modulates light stimulation-induced seizures in the DG and CA1. **A**: Flowchart of the surgical procedure and light stimulation experiment for ChR2-EYFP mice. **B**: Fluorescence images illustrate the expression of ChR2-EYFP in the DG (left) and CA1 (right) regions of the hippocampus, with dashed boxes indicating the locations of the optical fibers. Scale bar: 200 μm. **C**: Whole-cell recordings show that 10 Hz light stimulation can induce action potentials at the corresponding frequency in virus-infected neurons. **D**: Typical electroencephalograms (EEGs) and power spectrogram recordings during behavioral seizures received DG optogenetic light stimulation. The control EEG trace was collected from mice that received DG optogenetic light stimulation yet exhibited no seizure activity. **E**-**H**: The number of stimuli required for light stimulation of DG and CA1 principal neurons to induce seizures during diurnal and nocturnal phases. Statistical analysis was performed using the Mann-Whitney U test, with *** P < 0.001. All data are presented as mean ± SEM, and the number of mice used is indicated in each graph (ZT6 in DG: light orange; ZT 14 in DG: dark orange; ZT6 in CA1: light blue; ZT 14 in CA1: dark blue)

Pilocarpine-induced seizures predominantly originate in the hippocampus, which consists of several distinct subregions, including the dentate gyrus (DG), CA1, CA2, CA3, and subiculum. Among these subregions, the DG and CA1 have been the focus of extensive research concerning their roles in seizure activity, revealing notable differences in the electrophysiological characteristics of neurons within these areas. To investigate the contributions of these regions to the circadian rhythm of seizures, we induced seizures in the DG and CA1 using an optogenetic virus, pAAV2/9-CaMKIIα-hChR2(H134R)-EYFP (Figs. 2 A-B). A blue laser was utilized to activate different hippocampal regions in mice. As the number of stimulation sessions increased, significant epileptic seizures and epileptiform discharges were observed in both brain regions during both the nocturnal and diurnal phases (Fig. 2D). In the DG region, fewer stimuli were required to induce seizures at night, with a non-significant trend compared with the diurnal phase (Figs. 2E-F). Conversely, in the CA1 region, the number of stimuli necessary to induce seizures during the nocturnal phase was significantly higher than that required during the diurnal phase (Figs. 2G-H). Optogenetic stimulation of the CA1 region elicited significantly more generalized convulsive status epilepticus (GS) during the diurnal phase (ZT6: 11.5 ± 0.9 seizures) compared with the nocturnal phase (ZT14: 6.8 ± 1.1 seizures; unpaired t-test, *P* = 0.005). In contrast, GS frequency did not differ significantly between the diurnal and nocturnal time points upon DG stimulation (ZT6: 13.3 ± 2.1 seizures vs. ZT14: 12.9 ± 1.2 seizures; unpaired t-test, *P* = 0.88). Notably, no mortality was observed in any mouse receiving light stimulation targeting either the CA1 or DG subregion throughout the experimental period. These findings suggest that the DG region exhibits a lower seizure threshold during the nocturnal phase compared to the diurnal phase, while the CA1 region demonstrates a higher seizure threshold during the nocturnal phase. The modulation of seizure threshold by the circadian rhythm in the CA1 region parallels that observed with pilocarpine administration, indicating that the CA1 region may play a crucial role in mediating the circadian rhythm modulation of seizure activity.

### The circadian rhythm differently regulates gene expression in DG and CA1

**Fig. 3:**
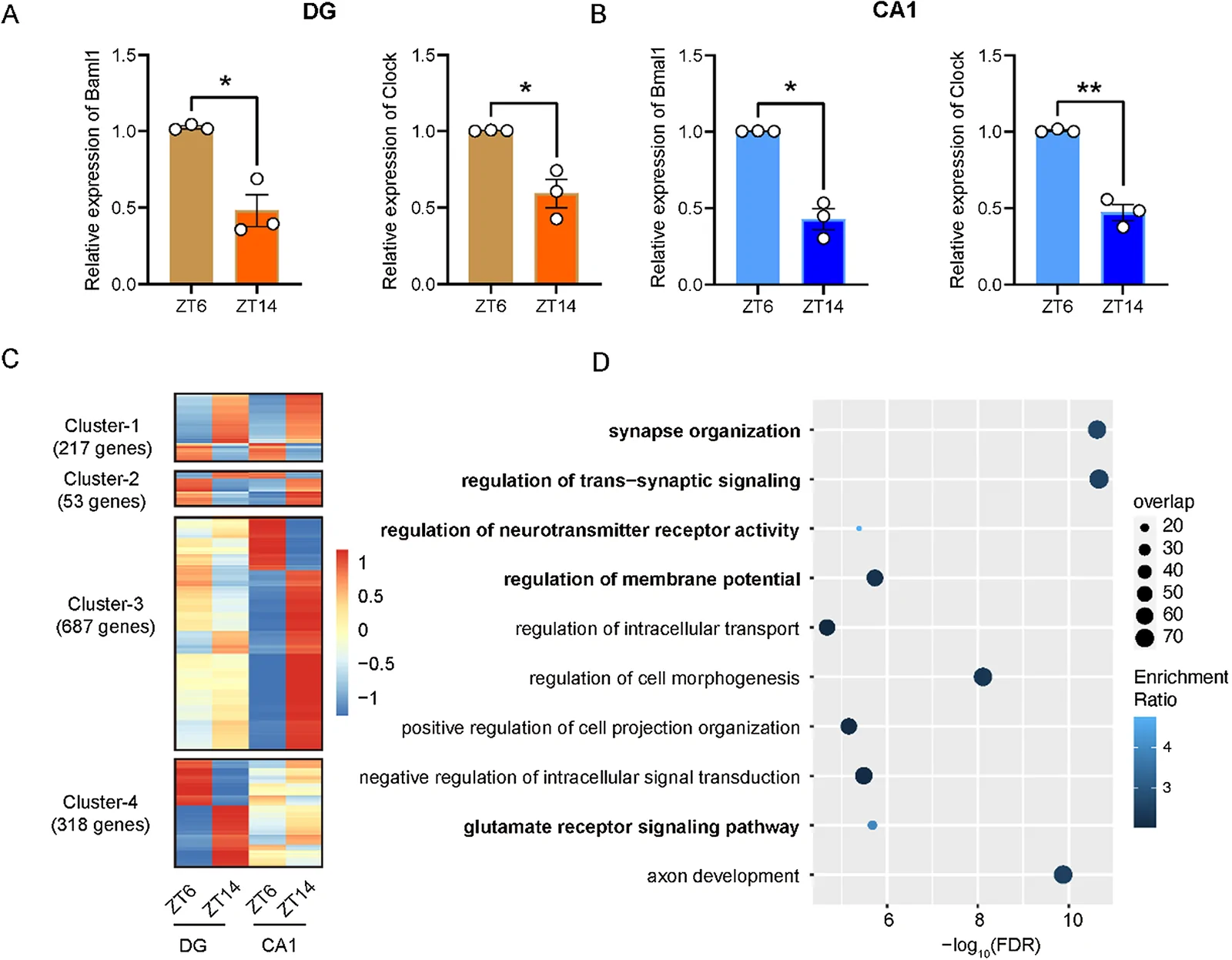
The circadian regulates gene expression in DG and CA1. **A**: Relative expression levels of Bmal1 and Clock in the DG at ZT6 and ZT14. **B**: Relative expression levels of Bmal1 and Clock in the CA1 at ZT6 and ZT14. **C**: Clustering analysis of differentially expressed genes (DEGs) identified through comparison between ZT6 and ZT14 conditions in the DG and CA1 regions. Cluster 1 represents genes that show consistent directional changes in both the CA1 and DG regions. Cluster 2 includes genes that exhibit opposite directional changes between the CA1 and DG regions. Cluster 3 consists of genes that are differentially expressed only in the CA1 region, while Cluster 4 contains genes that are differentially expressed only in the DG region. **D**: Top ten Gene Ontology (GO) terms enriched in genes from Cluster 2, 3 and 4. Each experimental group contained 3 mice

To investigate the mechanisms by which circadian modulation affects the seizure threshold in the dentate gyrus and CA1, we first compared the transcript shifts observed in the hippocampus, specifically focusing on Circadian Locomotor Output Cycles Kaput (Clock) and Brain and Muscle ARNT-Like 1 (Bmal1). We found a significant overlap in the expression of the core clock genes Clock and Bmal1 in both the DG and CA1 regions (Fig. 3AB). This result is consistent with the pattern of Zbtb14 protein expression observed in the DG and CA1 regions [25].

To capture the early transcriptional changes associated with circadian rhythms in the DG and CA1 region, tissues from the DG and CA1 were flash-frozen within one hour of collection. Poly(A) + RNA sequencing was conducted using an Illumina platform and mapped to the Mus musculus mm10 UCSC genome annotation. We identified 1,275 genes whose expression is regulated by circadian rhythms in either DG or the CA1 region. Among these, 217 genes exhibited shifts in expression in the same direction in both DG and CA1, while 53 genes shifted in opposite directions. Additionally, 687 genes shifted in CA1 but not in DG, and 318 genes shifted in DG but not in CA1 (Fig. 3C). We subsequently focused on the genes that displayed differential shifts between DG and CA1. The top Gene Ontology (GO) terms enriched for these genes included synapse organization, regulation of trans-synaptic signaling, regulation of synapse structure or activity, regulation of neurotransmitter receptor activity, regulation of membrane potential, glutamate receptor signaling pathway, and axon development (Fig. 3D). These findings suggest that synaptic transmission and intrinsic excitability may be modulated differently by circadian rhythms in DG and CA1, contributing to the differential modulation of seizure thresholds in these regions.

### The circadian rhythm differently modulates the excitatory/inhibitory balance of principal neurons in DG and CA1

**Fig. 4:**
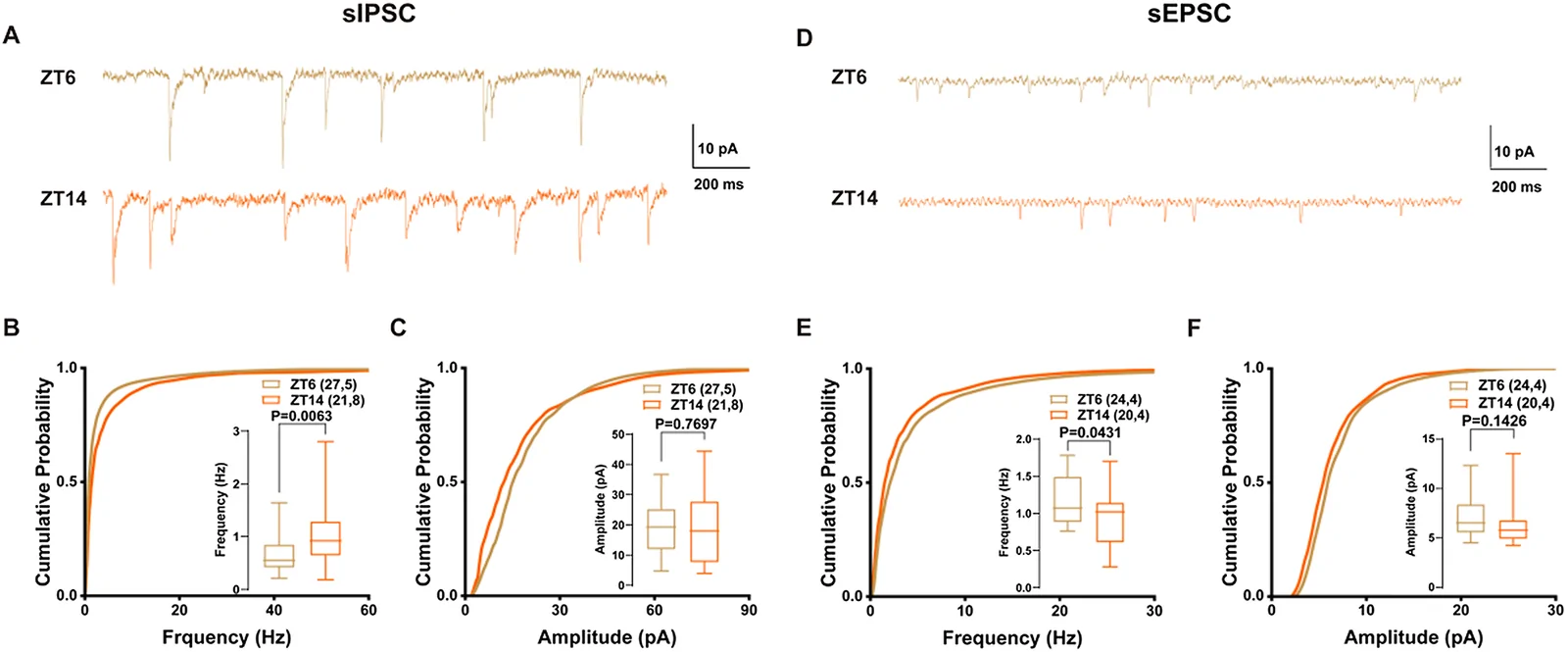
Different circadian regulation of inhibitory and excitatory synaptic transmission in dentate gyrus granule cells. (**A**) Representative spontaneous inhibitory postsynaptic current (sIPSC) traces recorded from DG granule cells (DGGCs) at ZT6 and ZT14. (**B**) Cumulative frequency distribution (Kolmogorov-Smirnov test, P < 0.05) and mean frequency (t-test) of sIPSCs in DGGCs at ZT6 vs. ZT14. (**C**) Cumulative amplitude distribution (Kolmogorov-Smirnov test, P < 0.05) and mean amplitude (t-test) of sIPSCs in DGGCs at ZT6 vs. ZT14. (**D**) Representative spontaneous excitatory postsynaptic current (sEPSC) traces recorded from DGGCs at ZT6 and ZT14. (**E**) Cumulative frequency distribution (Kolmogorov-Smirnov test, P < 0.05) and mean frequency (unpaired t-test) of sEPSCs in DGGCs at ZT6 vs. ZT14. (**F**) Cumulative amplitude distribution (Kolmogorov-Smirnov test, P < 0.05) and mean amplitude (unpaired t-test) of sEPSCs in DGGCs at ZT6 vs. ZT14. Each insert (in figure **B**,**C**,**E**,**F**) displays sample sizes formatted as (cells, mice)

We first investigated the circadian modulation of excitatory and inhibitory synaptic inputs to the principal neurons in the DG and CA1 regions of the hippocampus using brain slice patch-clamp techniques. Initially, we recorded the spontaneous inhibitory postsynaptic current (sIPSC) in DG granule cells (DGGC) (Fig. 4A). In contrast to the diurnal phase, the frequency of spontaneous inhibitory postsynaptic currents (sIPSCs) in DG granule cells (DGGCs) was significantly elevated during the nocturnal phase (Fig. 4B). No significant intergroup difference was detected in sIPSC amplitude via unpaired t-test, despite a divergent amplitude distribution confirmed by the Kolmogorov–Smirnov test (Fig. 4C). Additionally, we recorded spontaneous excitatory postsynaptic currents (sEPSC) in DGGC of the hippocampus. Our findings revealed that, in comparison to the diurnal phase, the frequency of sEPSC in DGGC during the nocturnal phase was significantly reduced (-Figure 4E), whereas the amplitude remained unchanged (Fig. 4F). These results suggest that DGGC exhibit a relatively low excitatory/inhibitory ratio during the nocturnal phase.

**Fig. 5:**
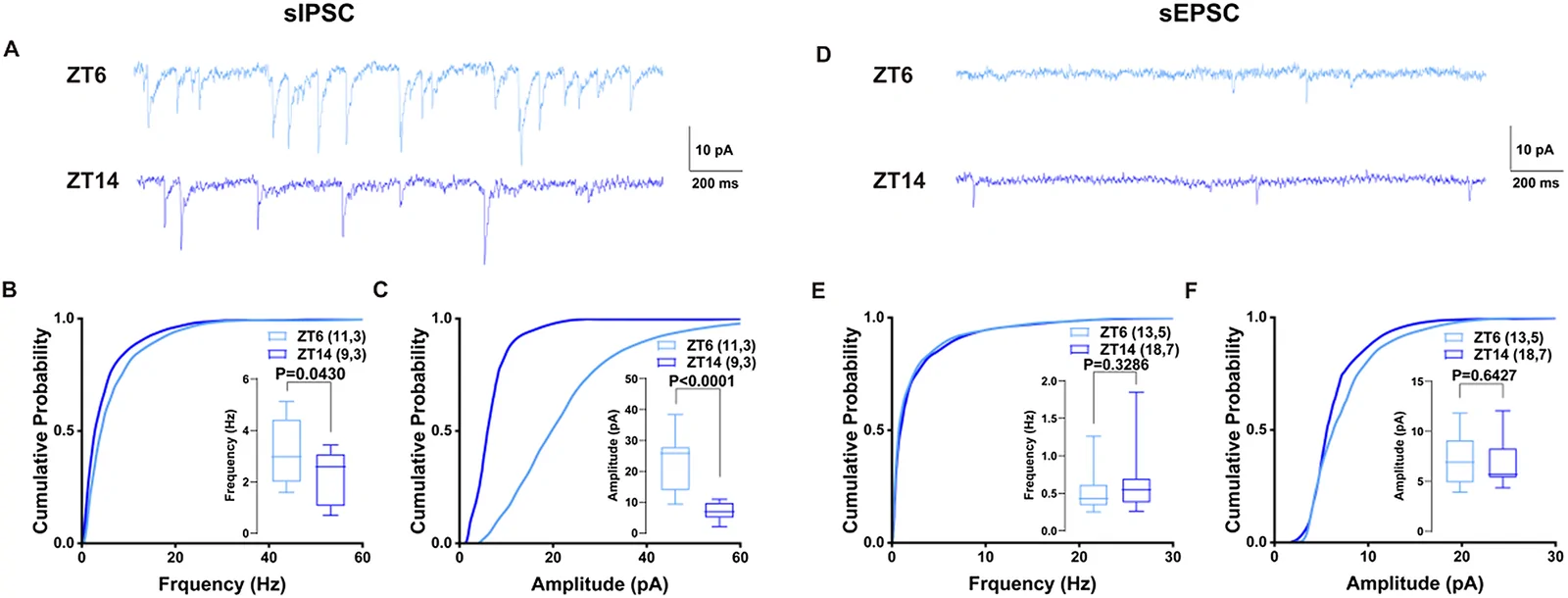
Different circadian modulation of inhibitory and excitatory synaptic transmission in CA1 pyramidal neurons. (**A**) Representative spontaneous inhibitory postsynaptic current (sIPSC) traces recorded from CA1 pyramidal neurons at ZT6 and ZT14. (**B**) Cumulative frequency distribution (Kolmogorov-Smirnov test, P < 0.05) and mean frequency (t-test) of sIPSCs in CA1 pyramidal neurons at ZT6 vs. ZT14. (**C**) Cumulative amplitude distribution (Kolmogorov-Smirnov test, P < 0.05) and mean amplitude (t-test) of sIPSCs in CA1 pyramidal neurons at ZT6 vs. ZT14. (**D**) Representative spontaneous excitatory postsynaptic current (sEPSC) traces recorded from CA1 pyramidal neurons at ZT6 and ZT14. (**E**) Cumulative frequency distribution (Kolmogorov-Smirnov test, P > 0.05) and mean frequency (unpaired t-test) of sEPSCs in CA1 pyramidal neurons at ZT6 vs. ZT14. (**F**) Cumulative amplitude distribution (Kolmogorov-Smirnov test, P < 0.05) and mean amplitude (unpaired t-test) of sEPSCs in CA1 pyramidal neurons at ZT6 vs. ZT14. Each insert (in figure B,C,E,F) displays sample sizes formatted as (cells, mice)

In our investigation of synaptic transmission within CA1 pyramidal neurons, we observed that the frequency of spontaneous inhibitory postsynaptic currents (sIPSCs) received by CA1 pyramidal neurons during the nocturnal phase demonstrated a significant increase relative to the diurnal phase (Fig. 5B), and the amplitude of sIPSCs also showed a significant elevation (Fig. 5C). Conversely, the frequency and amplitude of spontaneous excitatory postsynaptic currents (sEPSCs) during the nocturnal phase did not exhibit significant changes when compared to the diurnal phase via unpaired t-test, despite a divergent amplitude distribution confirmed by the Kolmogorov–Smirnov test (Fig. 5D-F). These findings suggest that CA1 pyramidal neurons maintain a relatively high excitation/inhibition ratio during the nocturnal phase, which aligns with existing literature [13].

### The circadian rhythm differently influences the intrinsic excitability of dentate gyrus granule cells (DGGC) and CA1 pyramidal neurons

**Fig. 6:**
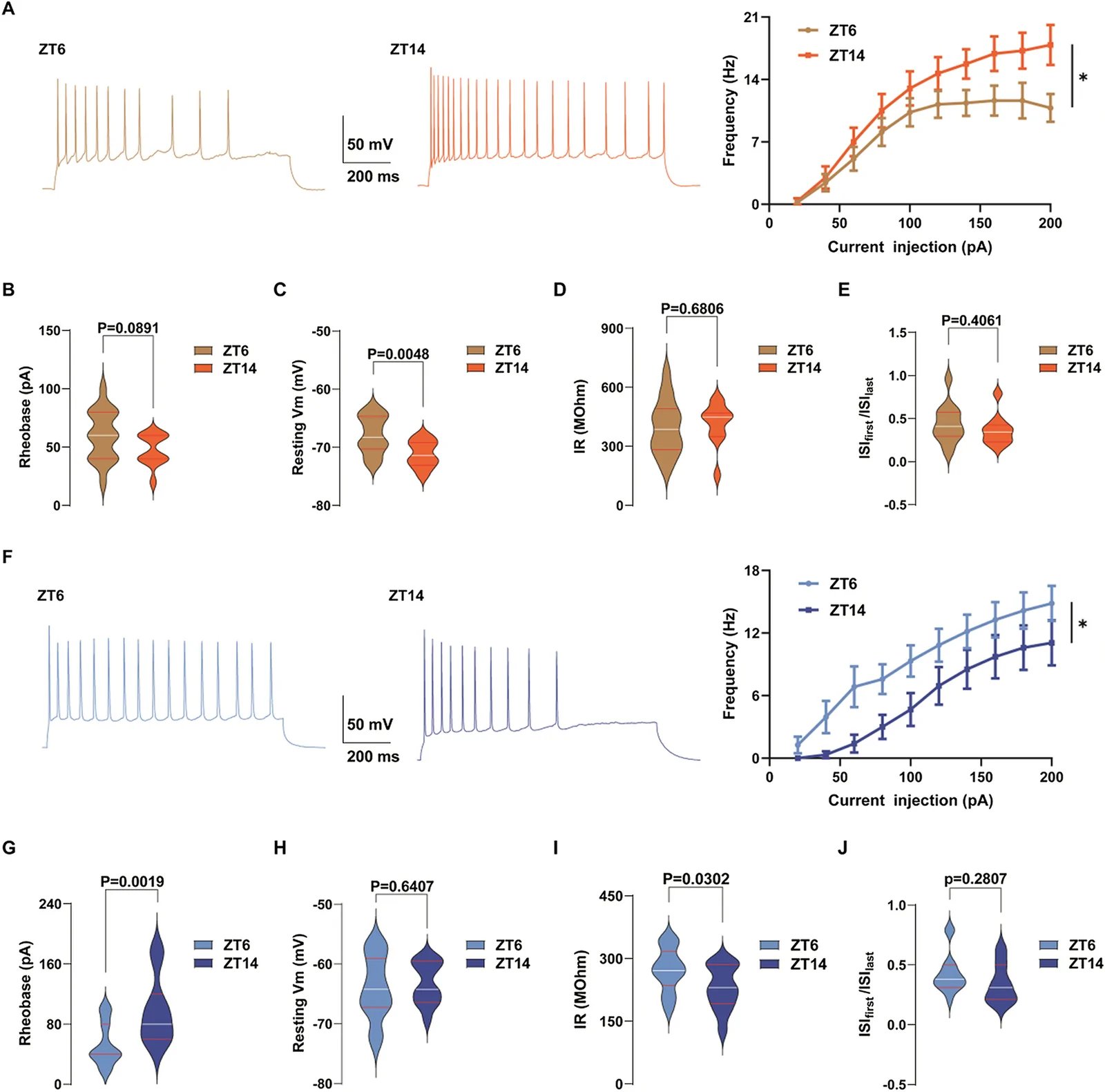
Opposing circadian modulation of intrinsic excitability in DG and CA1. (**A**) Representative firing responses of dentate gyrus granule cells (DGGCs) to identical current injections. Right: Current-step-evoked firing frequencies showed reduced spiking at ZT6. n = 19 from 4 mice (ZT6), 12 from 4 mice (ZT14); two-way ANOVA (ZT6 vs. ZT14 interaction): F(1,272) = 20.55, **P < 0.0001. (**B**) No circadian difference in rheobase, t-test, P > 0.05. (**C**) Resting membrane potential (Vm) was depolarized at ZT6, t-test, P < 0.05. (**D**) No circadian difference in input resistance (IR), unpaired t-test, P > 0.05. (**E**) No circadian difference in ISIfirst/ISIlast ratio, t-test: t = 0.848, P > 0.05 (NS). (**F**) Representative firing responses of CA1 pyramidal neurons (CA1PYs) to identical current injections. Right: Current-step-evoked firing frequencies showed reduced spiking at ZT14. n = 19 from 5 mice (ZT6), 15 from 7 mice (ZT14); two-way ANOVA (ZT6 vs. ZT14 interaction): F(1,320) = 28.08, P < 0.0001. (**G**) Rheobase increased at ZT14, t-test, P < 0.05. (**H**) No circadian difference in resting Vm, t-test, P > 0.05. (**I**) Input resistance (IR) was higher at ZT6, t-test, P < 0.05. (J) No circadian difference in ISIfirst/ISIlast ratio, t-test, P > 0.05

Then we investigate circadian rhythms modulation of the intrinsic excitability of DGGC and CA1 pyramidal neurons. We conducted recordings of the intrinsic excitability of principal neurons in the dentate gyrus (DG) and CA1 regions of the hippocampus. Our findings indicate that when the DGGC are subjected to identical current stimulation, they exhibit a higher firing frequency during the nocturnal phase (Fig. 6A), suggesting an elevated level of intrinsic excitability at this time. Further analysis demonstrated a notable increase in passive excitability during the nocturnal phase, characterized by a more hyperpolarized resting membrane potential (RMP) (Fig. 6C), and input resistance (IR) remained unchanged (Fig. 6D), and a reduced rheobase, which is the current necessary to reach the firing threshold (Fig. 6B). Concurrently, there were no significant alterations in the adaptation of continuous firing in DGGC (Fig. 6E). In contrast, the intrinsic excitability of CA1 pyramidal neurons is modulated differently by circadian rhythms compared to DGGC. Under the same current stimulation, CA1 pyramidal neurons display a lower firing frequency during the nocturnal phase (Fig. 6F), primarily attributable to their reduced cell input resistance (Fig. 6I) and elevated rheobase (Fig. 6G) at this time. Additionally, there were no significant changes observed in the resting potential or the adaptation of continuous firing in CA1 pyramidal cells. These results suggest that the intrinsic excitability of DGGC and CA1 pyramidal cells is regulated distinctly by circadian rhythms.

## Discussion

Here, we observe that the circadian rhythm differentially modulates the seizure threshold in the hippocampal dentate gyrus and CA1 regions. The excitatory and inhibitory synaptic transmissions, along with the intrinsic excitability of principal neurons in the DG and CA1, are influenced in opposing ways by the circadian rhythm. Moreover, intrinsic excitability may play a crucial role in the circadian modulation of seizure threshold in both the DG and CA1 regions.

The circadian rhythm is regulated by a series of biological clock proteins. Two critical components, Clock and Bmal1, form heterodimers that facilitate gene transcription. The proteins PER1/2 (Period 1/2), CRY1/2 (Cryptochrome 1/2), and Rev-erbα are involved in the positive and negative regulation of circadian oscillations mediated by Bmal1 and Clock [26, 27]. Rhythm-related genes can influence synaptic transmission and neuronal excitability in various ways, leading to distinct effects on epileptic seizures. Specifically, the knockout of the *Clock* gene has been shown to diminish inhibitory synaptic transmission in cortical pyramidal cells, which can result in seizures in sleeping mice [28]. Similarly, the knockout of the *Bmal1* gene lowers the seizure threshold and promotes seizure activity [8, 29]. This anticonvulsant effect is likely attributable to the increased excitability of dentate gyrus granule cells resulting from *Bmal* knockout [12]. Furthermore, *Rev-erbα* knockout can enhance GABAergic signaling by decreasing the recovery of the inhibitory neurotransmitter GABA in the dentate gyrus of the hippocampus, thereby elevating the seizure threshold and producing antiepileptic effects [9]. Most of these studies have knocked out the circadian protein throughout the whole brain. Recently, targeted deletion of *Bmal1* in SCN neurons also exacerbated seizures and diminished hippocampal GABAergic efficacy [5]. Temporal lobe epilepsy is classified into three subtypes: mesial temporal lobe epilepsy (MTLE), lesional temporal lobe epilepsy (LTLE), and neocortical temporal lobe epilepsy (NTLE). These subtypes exhibit distinct circadian rhythms [30]. Furthermore, MTLE shows two peak periods, occurring between 07:00–08:00 and 16:00–17:00 [31–33]. In this study, we demonstrate that circadian rhythms differentially modulate seizure thresholds in the dentate gyrus and CA1 regions, suggesting that the two peaks of MTLE may originate in different regions of the hippocampus.

DG and CA1 are both important regions for seizures. The dentate gyrus (DG) serves as a physiological gate [34]. After status epilepticus (SE), numerous pathological changes occur in the DG, including mossy fiber sprouting and a shift in excitatory/inhibitory (E/I) balance. These changes may contribute to epileptogenesis [11]. Similarly, CA1 also undergoes pathological alterations, such as neuronal loss [35]. Furthermore, transplanting inhibitory neurons into CA1 can help control seizures and abnormal behaviors [36, 37]. However, reprogramming reactive glial cells into interneurons in both the DG and CA1 has been shown to reduce seizures [38, 39]. In our study, we found that the modulation of seizure threshold in CA1 by circadian rhythm is similar to the effects observed with the administration of pilocarpine, suggesting that CA1 may be the most sensitive region for seizures in mice. Recent studies have demonstrated that the antiepileptic drug levetiracetam exerts beneficial effects on synaptic transmission and structural plasticity in the CA1 hippocampal region, while showing no significant impact on the impaired neurogenesis or aberrant mossy fiber sprouting observed in the DG of pilocarpine-induced SE rat models [40]. These findings align consistently with our experimental observations.

Synaptic transmission and intrinsic excitability are both crucial for seizure control. Antiseizure medications primarily target GABA (such as benzodiazepines), glutamate transmission (including perampanel and tiagabine), and sodium channels (like phenytoin, carbamazepine, and lamotrigine). However, there is significantly less focus on voltage gated potassium channel modulators, such as retigabine. Mutations in voltage gated potassium channels have been implicated in several conditions, including various epilepsies, and proved to play a crucial role in epilepsy treatments [41–43]. The potassium channel is a determining factor for membrane potential and membrane input resistance [44], which is essential for intrinsic excitability. Activation of potassium channels reduces firing frequency and decreases the efficiency of excitatory inputs [45]. Our findings indicate that intrinsic excitability plays a pivotal role in the circadian modulation of seizure thresholds. Antiseizure medications that target voltage gated potassium channels may have a beneficial effect on circadian rhythm-related seizures, such as those seen in temporal lobe epilepsy (TLE).

There are several limitations in this study. (1) This study only characterized acute pilocarpine-evoked seizures rather than the complete chronic pilocarpine model featuring recurrent spontaneous seizures after prolonged status epilepticus. The circadian regulatory patterns of seizure susceptibility observed under acute pharmacological stimulation may not recapitulate those of chronic spontaneous seizures in rodents or epileptic patients, as chronic epilepsy involves irreversible hippocampal neurodegeneration, gliosis and long-term network remodeling that fundamentally reshapes seizure circadian control. (2) Quantitative analyses only included overt stage 4 and stage 5 convulsive seizures accompanied by clear behavioral motor manifestations. Subclinical, non-convulsive hippocampal epileptiform discharges without visible convulsive behaviors were not systematically captured or quantified in this study, which may underestimate the total burden of hippocampal ictal activity across diurnal and nocturnal phases. (3) The conclusions drawn here are based on ICR mice. The circadian rhythm modulation in rats and humans may differ from that in ICR mice. (4) The CA2 region and subiculum may also play important roles in epileptogenesis [46–49]. The intrinsic excitability of CA2 pyramidal cell is increased in epilepsy mice and chemogenetic inhibition of CA2 PCs reduces the frequency of spontaneous seizures [48]. Subicular pyramidal neurons may also act as a key switch controlling drug-resistant temporal lobe epilepsy [50]. Additionally, excitatory subicular pyramidal neurons, which exhibit subregional heterogeneity, can bidirectionally modulate seizures in temporal lobe epilepsy [51]. Therefore, further investigation into the circadian rhythm modulation of the CA2 region and subiculum is warranted. (5) The circadian rhythm can regulate the expression of numerous genes. However, the detailed molecular mechanisms underlying circadian rhythm modulation in the dentate gyrus and CA1 region still require further exploration.

Overall, we demonstrate that circadian rhythms differentially modulate seizure thresholds, as well as excitatory and inhibitory synaptic transmissions and intrinsic excitability in the dentate gyrus and CA1 regions. In particular, the circadian modulation of intrinsic excitability in the CA1 region may be a key factor underlying the circadian rhythms observed in hippocampal seizures, which could have therapeutic implications for understanding the circadian patterns associated with temporal lobe epilepsy.

## Supplementary Information

Below is the link to the electronic supplementary material.

Supplementary Material 1

## Data Availability

The gene expression datasets generated and analysed during the current study are available in the Gene Expression Omnibus repository, https://www.ncbi.nlm.nih.gov/geo/query/acc.cgi? acc=GSE317534. The other datasets generated during and/or analysed during the current study are available from the corresponding author on reasonable request.
